# Beyond the Usual Suspects: Clinical and Pathological Insights From a Rare Case of Light Chain (AL) Amyloidosis in a Filipino Patient

**DOI:** 10.7759/cureus.88195

**Published:** 2025-07-17

**Authors:** Gilbert J Cabataña, Jude P Cebrecus, Susie Ponce, Albert L Rafanan, Gerard Saranza

**Affiliations:** 1 Neurology, Chong Hua Hospital, Cebu, PHL; 2 Internal Medicine, Chong Hua Hospital, Cebu, PHL

**Keywords:** amyloidosis, congo red staining, filipino, light chain amyloidosis, monoclonal plasma cell disorder, peripheral neuropathy

## Abstract

Systemic light chain (AL) amyloidosis is a rare, life-threatening disorder caused by the misfolding and deposition of monoclonal immunoglobulin light chains as amyloid fibrils in various organs, leading to progressive dysfunction. We report the case of a 58-year-old Filipino male who presented with a four-month history of painful paresthesia, progressive neuropathy, significant weight loss, and autonomic symptoms, including orthostatic hypotension and gastrointestinal dysmotility. Initial workup revealed a length-dependent axonal and demyelinating polyneuropathy. Chest imaging showed diffuse micronodules and lymphadenopathy, prompting further investigation. Histopathological analysis of lymph node tissue with Congo red staining confirmed amyloid deposition, while bone marrow immunohistochemistry demonstrated monoclonal kappa light chain expression and markers consistent with lymphoplasmacytic lymphoma. The patient’s multisystemic involvement, including peripheral and autonomic neuropathy, exemplifies the protean clinical manifestations of AL amyloidosis and the diagnostic challenges it poses, particularly in resource-limited settings. Early recognition is crucial, as delayed diagnosis often results in irreversible organ damage and poor prognosis for those presenting with neuropathy. Treatment, adapted from multiple myeloma protocols, centers on eliminating the amyloidogenic clone using bortezomib, cyclophosphamide, and dexamethasone, although access to newer agents like daratumumab remains limited. This case underscores the need for heightened clinical suspicion, multidisciplinary collaboration, and improved diagnostic infrastructure to optimize outcomes for patients with AL amyloidosis in the Philippines and similar settings.

## Introduction

Systemic amyloidosis is a rare disease resulting from the misfolding of pathogenic proteins, leading to the extracellular deposition of insoluble amyloid fibrils in multiple organ systems [1,2]. The nonspecific and varied clinical manifestations of this disorder often lead to a delayed diagnosis. Light chain (AL) amyloidosis - an acquired, ultra‐rare subtype - is associated with significant morbidity and mortality. Despite an estimated global crude annual incidence of approximately 10.44 cases per million [3,4], epidemiologic data are limited internationally, especially in the Philippines, where only a few cases have been reported [5]. Accurate diagnosis requires patient and physician education to advance research and optimize clinical practice [1,2,4].

## Case presentation

A 58‐year‐old Filipino male from Cebu, with no known comorbidities, presented with a four‐month history of progressively painful paresthesia. The sensory disturbances initially involved the feet and later extended to all extremities. He first sought evaluation from a rheumatologist, who initiated treatment with pregabalin, yielding only partial relief. Due to the persistence and aggravation of symptoms, a referral was made to a neurologist. An electrodiagnostic evaluation using electromyography and nerve conduction velocity (EMG-NCV) revealed a length‐dependent axonal and demyelinating polyneuropathy. Other relevant diagnostic workups were unrevealing (Tables 1-2).

**Table 1:** Diagnostic imaging CT: computed tomography; bpm: beats per minute; EF: ejection fraction; GLS: global longitudinal strain

Test/imaging	Findings/results
Chest X-ray	Normal heart and lungs; atherosclerotic aorta
CT chest (plain)	Centrilobular nodules (inflammatory etiology); subsegmental atelectasis/fibrosis in the right middle lobe and lung bases
2D Echo with GLS	Concentric left ventricular hypertrophy, EF 60%; speckled myocardium; average global peak longitudinal strain: -20.17%; mild tricuspid regurgitation; minimal pericardial effusion
24-hour Holter	Sinus rhythm (avg. 71 bpm); rare premature atrial/ventricular complexes; no ventricular tachycardia/fibrillation

**Table 2:** Laboratory diagnostics AFP: alpha-fetoprotein; ALT: alanine transaminase; Baso: basophils; CA19-9: carbohydrate antigen 19-9; CBC: complete blood count; CEA: carcinoembryonic antigen; Eos: eosinophils; HCT: hematocrit; Hgb: hemoglobin; Lymph: lymphocytes; Mono: monocytes; PLT: platelets; PSA: prostate-specific antigen; Seg: segmented neutrophils; WBC: white blood cell count

Parameter measured	Reference range	Results
CBC		
WBC	4.8-10.8 × 10^3^/μL	6 × 10^3^/μL
Hgb	14-18 g/dL	9.3 g/dL
HCT	42-52%	27%
PLT	130-400 × 10^3^/μL	360 × 10^3^/μL
Seg	40-74%	61%
Lymph	19-48%	20.7%
Mono	3.4-9%	12.5%
Eos	0-7%	4.8%
Baso	0-1.5%	1.0%
Metabolic panel		
HbA1c	<5.7%	4.8%
Creatinine	0.60-1.20 mg/dL	0.73 mg/dL
ALT	<42 U/L	15 U/L
Sodium	134-148 mEq/L	137 mEq/L
Potassium	3.3-5.3 mEq/L	3.8 mEq/L
Tumor markers		
PSA	0-4 ng/mL	0.19 ng/mL
AFP	0-7 IU/mL	<0.75 IU/mL
CA 19-9	0-37 U/mL	6.92 U/mL
CEA	0.14-6.5 ng/mL	1.76 ng/mL

**Figure 1:**
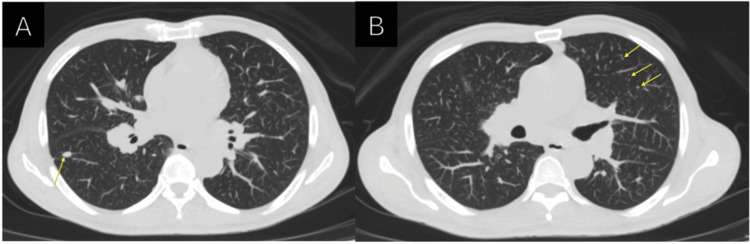
Axial view of the patient’s non-contrast computed tomography (CT) scan of the chest (A-B) Centrilobular micronodules (yellow arrows) are diffusely seen in both the upper and lower lobes of the lung.

In addition to the neuropathic symptoms, the patient experienced significant unintentional weight loss (approximately 10 kilograms), generalized fatigue, and a chronic, intermittent dry cough without hemoptysis over the following two months. Given the endemic nature of pulmonary tuberculosis in the region, a pulmonologist evaluated the patient. A chest CT scan with contrast demonstrated diffusely distributed micronodules and lymphadenopathy, prompting further investigation (Figure 1).

**Figure 2:**
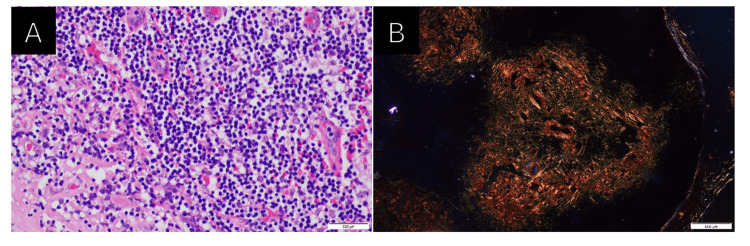
Histopathology of the cervical lymph node (A) High-power objective in hematoxylin and eosin stain showing aggregates of small- to medium-sized neoplastic lymphoid cells. (B) Special staining with Congo red showing apple-green birefringence in polarized light, signifying amyloid deposition.

**Figure 3:**
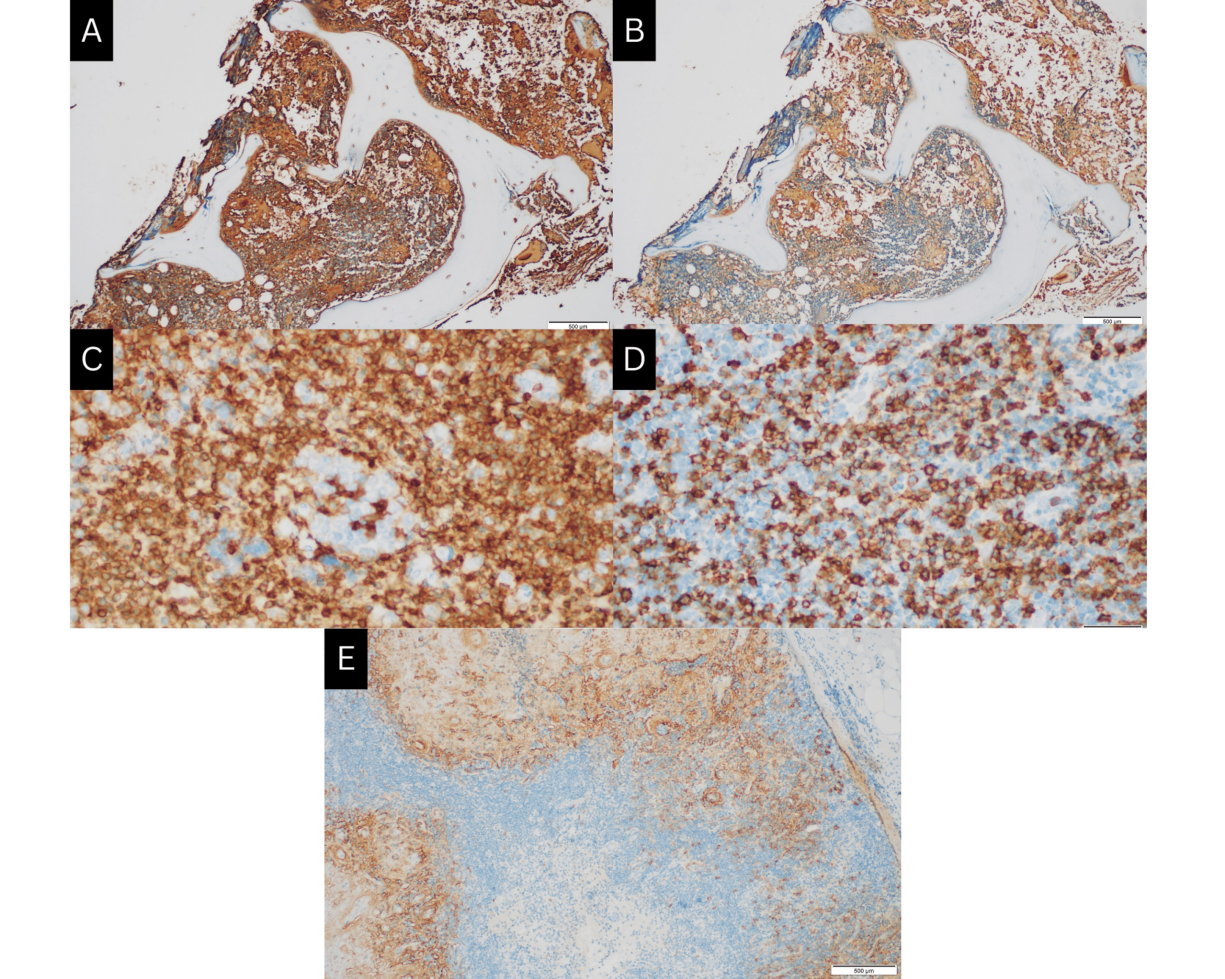
Immunohistochemistry of bone marrow and cervical lymph node (A) Bone marrow aspirate showing positive monoclonal expression for Kappa. (B) Bone marrow aspirate showing no significant expression of lambda. (C) Lymph node specimen positive for CD20 showing diffuse strong cytoplasmic membrane expression in neoplastic B-lymphoid cells. (D-E) Lymph node specimen positive for CD138 showing strong cytoplasmic membrane expression in plasma cells comprising 5-10% of marrow cellularity.

A biopsy of the cervical lymphoid tissue revealed nonspecific chronic lymphadenitis, thereby raising the suspicion of amyloidosis. Histopathological studies were subsequently undertaken, with Congo red staining confirming the presence of amyloid (Figure 2). Immunohistochemistry of the bone marrow aspirate showed positive monoclonal expression of the kappa light chain along with markers CD20 and CD138, findings that were compatible with a diagnosis of lymphoplasmacytic lymphoma (Figure 3). Notably, there was no family history of amyloidosis, and no relatives had been worked up for this condition.

As his illness progressed, the patient developed autonomic dysfunction manifesting as xerostomia, anhidrosis, dry eyes, constipation, erectile dysfunction, and syncopal episodes upon rapid postural changes. Exertional dyspnea also emerged. The patient is currently undergoing treatment with bortezomib, cyclophosphamide, and dexamethasone; however, he is unable to afford the recommended regimen, including daratumumab.

## Discussion

The pathogenesis of systemic amyloidosis involves the misfolding of proteins from their native α‐helical configuration into β‐pleated sheets, which are resistant to proteolysis. These misfolded proteins aggregate into amyloid fibrils that deposit extracellularly, ultimately leading to progressive organ dysfunction [6]. In AL amyloidosis - the most common form of primary systemic amyloidosis - a plasma cell dyscrasia occurs whereby a small clone of monoclonal plasma cells produces immunoglobulin light chains that form amyloid fibrils. These fibrils may infiltrate various organs such as the heart, kidneys, nerves, liver, lungs, and gastrointestinal tract, generating various clinical manifestations [7,8].

Four requirements must be met for a diagnosis of AL amyloidosis based on criteria established by the Mayo Clinic and the International Myeloma Group [1]: (a) the presence of an amyloid-related systemic syndrome; (b) positive amyloid staining by Congo red or the presence of amyloid fibrils on electron microscopy; (c) evidence that the amyloid is light chain-related; and (d) proof of a monoclonal plasma cell proliferative disorder.

Globally, AL amyloidosis is rare, and local data are particularly scarce. To date, only one case report from the Philippines, published in 2003, has detailed a case of primary systemic amyloidosis in a 52‐year‐old patient whose presentation included cardiac arrhythmia, with the diagnosis confirmed post-mortem [9]. Often, the initial nonspecific symptoms result in delayed diagnosis and contribute to irreversible organ damage, emphasizing the critical need for early recognition [2,10].

In AL amyloidosis, the heart is involved in approximately 75% of cases, the kidneys in 57%, the nerves in 22%, and the liver in 20%. Peripheral neuropathy, frequently an incidental finding during a routine examination, typically presents initially as a symmetric, small fiber neuropathy with length‐dependent loss of pain and temperature sensation in the distal extremities. Often, these symptoms involve larger nerve fibers, resulting in motor weakness and numbness. When neuropathy is the presenting feature, the average duration from symptom onset to diagnosis can range from 29 to 48 months [1,6,11]. Autonomic neuropathy - evident as orthostatic hypotension, urinary retention, and gastrointestinal dysmotility - is observed in about 65% of patients with neuropathy and may independently portend a worse prognosis [1,2,6,11]. Additionally, atypical neurological presentations such as cranial neuropathy, facial diplegia, multiple cranial neuropathies, or a picture resembling chronic inflammatory demyelinating polyneuropathy (CIDP) have also been reported [6].

The median survival for patients with AL amyloidosis presenting with neuropathy is reported to be between 25 and 35 months. Although neuropathy was once believed to be irreversible, emerging evidence indicates that early therapeutic intervention can result in the improvement of both peripheral and autonomic functions concomitant with disease regression [6]. Management strategies for AL amyloidosis are adapted from multiple myeloma protocols but modified to account for the patient’s susceptibility to treatment toxicity. The primary objective is to eliminate the amyloid precursor (the free light chain) through anti-clonal therapy, thereby improving organ function and prolonging survival [10,12]. Historically, treatment with melphalan and dexamethasone was common; however, recent protocols favor a four-drug induction regimen - comprising daratumumab, cyclophosphamide, bortezomib, and dexamethasone - as supported by the phase III ANDROMEDA trial [12]. Therapeutic response is measured in terms of hematologic improvement and organ-specific parameters, such as reductions in N-terminal pro-B-type natriuretic peptide (NT-proBNP) levels for cardiac involvement, urinary protein losses for renal involvement, and alkaline phosphatase levels for hepatic involvement [10,12]. Objective evaluations of neuropathy, such as the modified neuropathy impairment score +7 (mNIS+7) and nerve conduction studies, further aid in monitoring treatment efficacy [6,8].

Supportive care is integral to the management of AL amyloidosis. A multidisciplinary approach is essential to monitor and manage cardiac complications, peripheral and autonomic neuropathy, and treatment-related adverse effects. For neuropathic pain, gabapentinoids (e.g., gabapentin, pregabalin) or duloxetine may be beneficial. Autonomic symptoms, such as orthostatic hypotension, can be managed with dietary modifications (high salt intake), fludrocortisone, or compression stockings. In cases of cardiomyopathy, midodrine - an alpha-adrenergic agonist - may be utilized [7,8,10].

## Conclusions

This case highlights the diagnostic challenges associated with systemic amyloidosis, particularly AL amyloidosis, within a resource-limited setting such as the Philippines. The disease’s nonspecific clinical presentation and multisystem involvement necessitate a high index of suspicion and a comprehensive multidisciplinary diagnostic approach that includes histopathological confirmation and advanced immunological testing. Greater awareness and documentation of such cases are vital to improving epidemiological data, guiding clinical management, and fostering research into effective treatment strategies for systemic amyloidosis.
